# Response to “Comment on ‘Lessons from Toxicology: Developing a 21st-Century Paradigm for Medical Research’”

**DOI:** 10.1289/ehp.1611305

**Published:** 2016-05-01

**Authors:** Gill Langley

**Affiliations:** Research and Toxicology Department, Humane Society International, London, United Kingdom

In his letter, Greek correctly emphasizes the importance of theoretical foundations for understanding experimental observations. Our proposed human-specific paradigm for medical research and drug discovery does indeed rest on a strong theoretical, as well as empirical, basis.

Because of the longevity of the animal research paradigm, in medical studies it is often assumed that animal-based data are generally applicable to humans, overlooking the significance of the species barrier as described by Greek. It is crucial to be aware that rodents and humans diverged evolutionarily 65–85 million years ago (Kumar and Hedges 1998), allowing plenty of time for disparities to develop in structure and functionality at all levels of biological complexity. Thus, inherent differences between mouse and human genetic backgrounds, immune systems, and brain circuitry are limiting progress in understanding autism spectrum disorders (Muotri 2015). In Alzheimer’s disease research, too, underlying species variations in genetics, protein pathways, metabolism, pharmacology, and physiology are very challenging (Langley 2014).

The theory that inter- and intraspecies disparities confound extrapolation from other animals to humans is illustrated in many medical research fields, including stroke, motor neuron disease, Huntington’s disease, asthma, sepsis, and inflammatory disorders (Langley 2014). Although improvements could be made to the experimental design and methodology of animal studies, they remain inevitably flawed by the “insuperable species barrier” (Van Dam and De Deyn 2011), and better prospects for progress are becoming available through advanced techniques applied to reliable human-specific models.

Humans (and many diseases) are complex systems, so a systems biology framework will be vital to integrate human-specific data of different levels of biological complexity, and to enable a dynamic understanding of disease causes, pathophysiologies, and potential drug targets (Zou et al. 2013). Machine-based data mining that captures and represents scientific knowledge in a structured format is already contributing to the construction of human disease pathways and networks (Kodamullil et al. 2015). There are still challenges in handling “big data,” including improving the curation and means of visualizing and characterizing complex systems.

The reliability of any model can be considered in terms of its validity. Face validity addresses phenomenological similarities between a model and a human disease, but superficial similarities do not reliably imply the same underlying mechanisms. Moreover, most animal models only recapitulate limited pathophysiological aspects of human conditions. Construct validity does ask whether the model reflects the etiology and underlying biology of the human disease. However, the development of transgenic animal models based on human disease pathways can only be attempted after a pathway is already known to be clinically significant. Emerging human- and disease-specific *in vitro* models (such as those derived from human induced pluripotent stem cells) potentially offer a way forward, by enabling the discovery and detailed study of new human pathways and drug targets. Predictive validity asks whether a model reliably predicts what happens in humans, especially the effects of therapeutic interventions. This is a key factor in translational science and an obvious weakness in many animal studies, as shown by an analysis of 76 highly cited articles on several different animal species, published in seven high-impact scientific journals, which found that only 37% of the studies accurately predicted human outcomes (Hackam and Redelmeier 2006).

The scientific literature increasingly includes critical assessments of the validity of animal models, reflecting serious concerns about their reliability and predictive value for human outcomes (Akhtar 2015). Few animal models have been evaluated by systematic review and meta-analysis of their performance characteristics such as reproducibility, specificity, sensitivity, clinical relevance, or mechanistic basis, and those that have frequently have performed poorly (Pound and Bracken 2014). Recognition of the problems is the first, essential step to finding solutions. A wider understanding is needed, based on both theoretical and empirical foundations, of the failure of animal models in medical research and drug discovery, and the reasons for that failure. That recognition will help drive the development and implementation of a radically improved 21st-century research paradigm.
